# Understanding Hepatic Encephalopathy: Patient Knowledge, Adherence, and Barriers to Care in Cirrhosis

**DOI:** 10.7759/cureus.106215

**Published:** 2026-03-31

**Authors:** Noor Albusta, Ahmed Ali, Hussain Alrahma

**Affiliations:** 1 Internal Medicine, Beth Israel Lahey Health, Burlington, USA; 2 General Surgery, Salmaniya Medical Complex, Manama, BHR; 3 Medicine, Bahrain Government Hospitals, Manama, BHR; 4 Gastroenterology and Hepatology, Bahrain Government Hospitals, Manama, BHR

**Keywords:** barriers to care, hepatic encephalopathy, liver cirrhosis, medication adherence, patient knowledge

## Abstract

Introduction: Hepatic encephalopathy (HE) is a common and debilitating complication of liver cirrhosis, associated with impaired cognition, reduced quality of life, and frequent hospitalizations. Effective management requires adherence to medications such as lactulose and rifaximin; however, patient understanding and adherence remain suboptimal. This study aimed to assess patient knowledge of HE, medication adherence, and perceived barriers to care among patients with cirrhosis.

Methods: We conducted a cross-sectional study in Bahrain Government Hospitals between January 2022 and December 2024. Adult patients with liver cirrhosis were recruited during outpatient and inpatient encounters. A structured questionnaire assessed knowledge of HE, adherence to treatment, and barriers to care. Adherence and knowledge were assessed using self-reported measures. Factors associated with poor knowledge and non-adherence were analyzed using multivariable logistic regression.

Results: A total of 538 patients with cirrhosis were included. The mean age was 56 ± 13 years, and 312 (58.0%) were male. Only 214 patients (39.8%) demonstrated adequate knowledge of HE. While 402 patients (74.7%) were prescribed lactulose, only 279 (52.0%) reported good adherence. Rifaximin was prescribed in 409 patients (76.0%), with higher adherence rates at 74.3% (304 of 409 patients). The most common barriers to adherence included medication side effects (41.2%), lack of understanding of treatment purpose (38.5%), cost concerns (27.3%), and forgetfulness (24.1%). Patients with prior HE episodes had significantly higher knowledge scores (p<0.001). Poor adherence was independently associated with low educational level (OR 2.1, 95% CI 1.3-3.5), inadequate knowledge (OR 2.8, 95% CI 1.7-4.6), and lactulose-related side effects (OR 1.9, 95% CI 1.2-3.1).

Conclusion: Patient knowledge and adherence to HE therapy remain suboptimal, with significant barriers affecting effective management. Targeted patient education and strategies to improve the tolerability of therapy are essential to reduce HE-related morbidity.

## Introduction

Hepatic encephalopathy (HE) is a neuropsychiatric syndrome resulting from liver dysfunction and portal-systemic shunting, characterized by a wide spectrum of cognitive and motor disturbances ranging from subtle impairment to coma [1]. It represents a major complication of liver cirrhosis and is associated with increased morbidity, mortality, and healthcare utilization [2,3]. The pathogenesis of HE is multifactorial, with ammonia accumulation playing a central role alongside systemic inflammation, altered neurotransmission, and gut dysbiosis [4].

Recurrent HE significantly impairs quality of life and is a leading cause of hospital readmission among patients with cirrhosis [4]. Standard management focuses on reducing ammonia production and absorption, primarily through non-absorbable disaccharides such as lactulose and antibiotics such as rifaximin [1,4]. Current guidelines recommend lactulose as first-line therapy, with rifaximin added for secondary prophylaxis following an episode of overt HE [1,4]. Meta-analyses have demonstrated that combination therapy with rifaximin plus lactulose reduces mortality, recurrence of HE, and hospitalizations compared with lactulose alone [2,3].

Despite well-established treatment strategies, the effectiveness of therapy is highly dependent on patient adherence and understanding of the disease. Lactulose is frequently associated with gastrointestinal side effects such as diarrhea, bloating, and abdominal discomfort, which can negatively impact adherence [5]. Previous studies have shown that patients often have limited understanding of HE, including its triggers, prevention strategies, and treatment goals [5,6]. Poor knowledge and adherence have been associated with increased risk of HE recurrence and hospitalization [7-9]. Real-world data suggest that a substantial proportion of patients fail to refill medications after discharge, further increasing the risk of decompensation [9].

Barriers to effective management may include medication side effects, lack of understanding, cost, and healthcare access [5]. Data on patient knowledge and adherence to HE therapy in the Middle East are limited, and regional factors such as health literacy and healthcare systems may influence outcomes. Therefore, this study aimed to evaluate patient knowledge of HE, adherence to treatment, and barriers to care among patients with cirrhosis in Bahrain, and to identify factors associated with poor adherence.

## Materials and methods

Study design and setting

This cross-sectional study was conducted in Bahrain Government Hospitals between January 1, 2022 and December 31, 2024. Patients were recruited using a consecutive sampling approach during inpatient admissions and outpatient clinic visits. Ethical approval was obtained from the institutional review board, and informed consent was obtained from all participants.

Study population

Adult patients (≥18 years) with a diagnosis of liver cirrhosis were eligible for inclusion. Cirrhosis was defined based on clinical, laboratory, or radiological findings. Patients were included if they were able to provide informed consent or had a caregiver present. Exclusion criteria included acute liver failure, severe cognitive impairment precluding questionnaire completion, and inability to communicate in Arabic or English without translation.

Data collection

Data were collected using a structured, interviewer-administered questionnaire during inpatient and outpatient encounters. The questionnaire was developed based on current clinical guidelines and published literature on HE and cirrhosis management. It comprised multiple domains including demographic characteristics (age, sex, and educational level), clinical characteristics (etiology of cirrhosis and prior HE episodes), medication use (lactulose and rifaximin), adherence to therapy, knowledge of HE, and perceived barriers to care.

Educational level was recorded and categorized as no formal education/primary education, secondary education, or university-level education. For analytical purposes, a low educational level was defined as secondary education or below.

Assessment of knowledge

Knowledge of HE was assessed using a 10-item questionnaire developed based on current clinical practice guidelines (see Appendices). The questionnaire covered key domains including pathophysiology, common triggers (e.g., constipation, infection), recognition of symptoms, and the role of medications in prevention and treatment. Each correct response was assigned one point, yielding a total score ranging from 0 to 10. A score ≥7 was considered indicative of adequate knowledge.

Assessment of adherence

Medication adherence was assessed using self-reported responses. Patients were asked to estimate the proportion of time they took their prescribed medications as directed. Good adherence was defined as taking medication ≥80% of the time, while poor adherence was defined as taking medication <80%, consistent with established definitions in chronic disease management research [10].

Barriers to care

Barriers to medication adherence were assessed using predefined response options derived from existing literature. Patients were asked to identify factors contributing to non-adherence, including medication side effects, cost concerns, forgetfulness, lack of understanding of treatment purpose, and difficulty accessing medications. Multiple responses were permitted.

Statistical analysis

Continuous variables were expressed as mean ± standard deviation or median (interquartile range), and categorical variables as frequencies and percentages. Comparisons were performed using Student’s t-test or Mann-Whitney U test for continuous variables, and chi-square or Fisher’s exact test for categorical variables. Variables with p<0.10 in univariate analysis were included in multivariable logistic regression to identify independent predictors of poor adherence. Results were reported as odds ratios (ORs) with 95% confidence intervals (CIs). A p-value <0.05 was considered statistically significant. Analyses were performed using IBM SPSS Statistics for Windows, Version 28 (Released 2021; IBM Corp., Armonk, New York, United States).

## Results

Baseline characteristics

**Figure 1:**
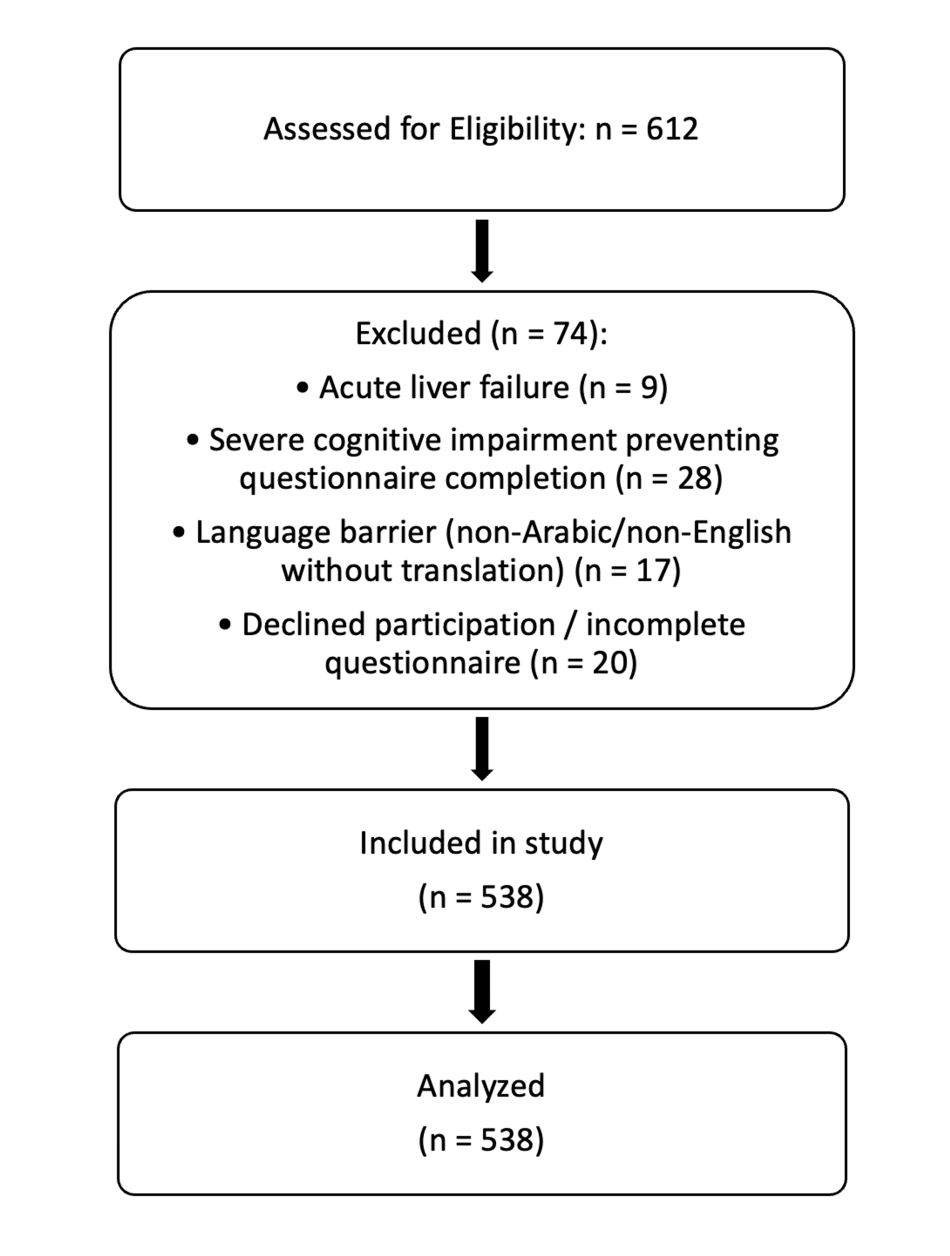
Flow Diagram of Patient Screening and Selection

A total of 538 patients with liver cirrhosis were included in the final analysis after screening and eligibility assessment (Figure 1).

The mean age was 56 ± 13 years, and 312 (58.0%) were male. The most common etiologies of cirrhosis were viral hepatitis (34.2%), non-alcoholic fatty liver disease (29.6%), and alcohol-related liver disease (18.7%). A total of 278 patients (51.7%) had a prior history of HE (Table 1).

**Table 1:** Baseline Characteristics of Patients with Cirrhosis NAFLD: Non-alcoholic fatty liver disease

Variable	Value
Age, years (mean ± SD)	56 ± 13
Male sex, n (%)	312 (58.0)
Etiology of cirrhosis, n (%)	
Viral hepatitis	184 (34.2)
NAFLD	159 (29.6)
Alcohol-related liver disease	101 (18.7)
Other	94 (17.5)
Prior hepatic encephalopathy, n (%)	278 (51.7)
Medication use, n (%)	
Lactulose	402 (74.7)
Rifaximin	409 (76.0)

Knowledge of HE

Only 214 patients (39.8%) demonstrated adequate knowledge of HE. Knowledge gaps were identified in multiple domains: 55.4% of patients were unaware of common triggers, 51.7% had poor recognition of early symptoms, and 47.3% did not understand the purpose of lactulose therapy. Patients with prior HE episodes had significantly higher knowledge scores (p<0.001).

Medication use and adherence

Among the study population, 402 patients (74.7%) were prescribed lactulose and 409 patients (76.0%) were prescribed rifaximin. Good adherence to lactulose was reported in 279 patients (52.0%), whereas adherence to rifaximin was higher at 74.3% (304 of 409 patients).

Barriers to adherence

**Figure 2:**
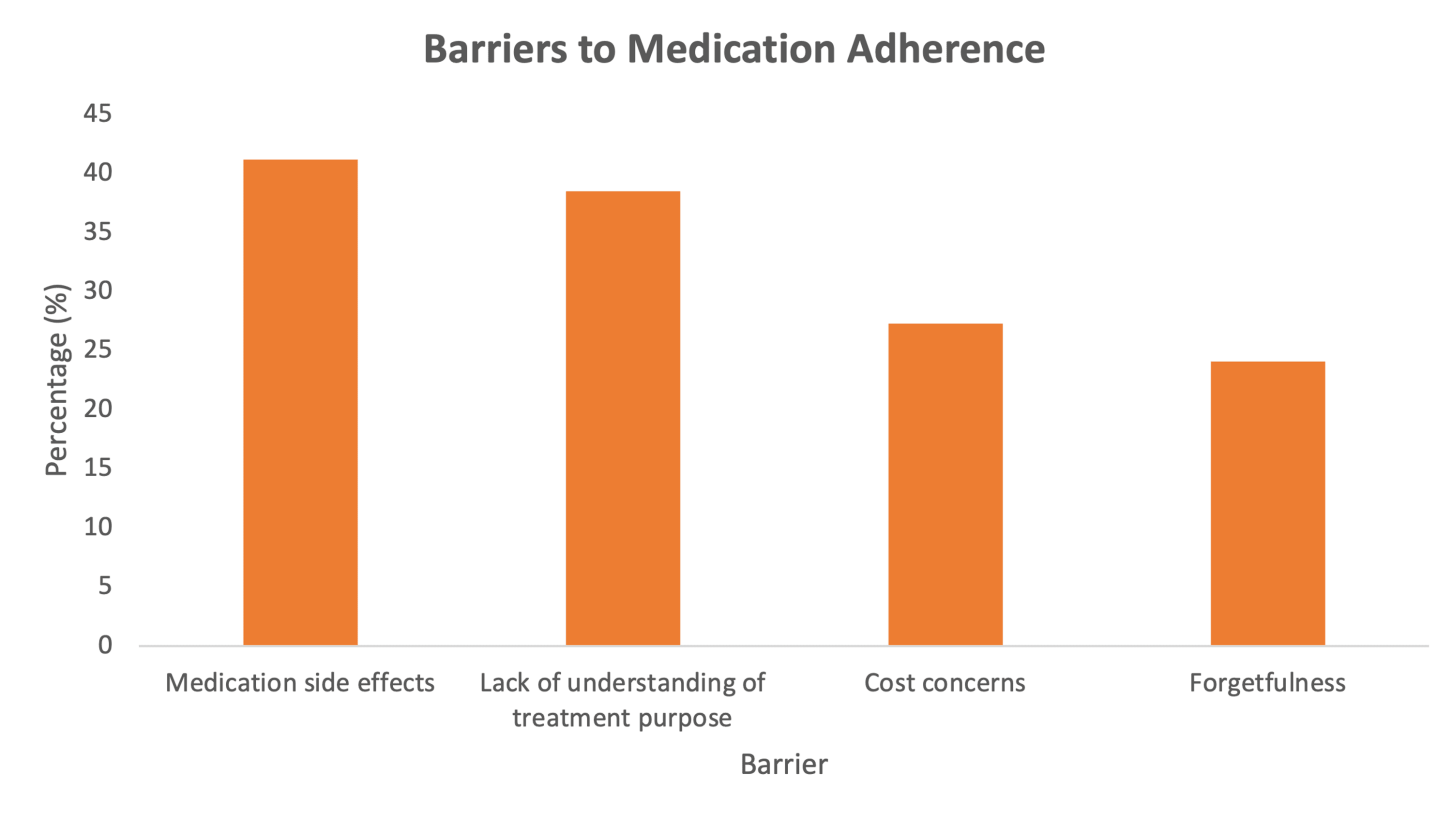
Patient-Reported Barriers to Hepatic Encephelopathy Treatment Adherence

The most commonly reported barriers to adherence included medication side effects (222, 41.2%), lack of understanding of treatment purpose (207, 38.5%), cost concerns (147, 27.3%), and forgetfulness (130, 24.1%). Lactulose-related side effects were the leading contributor to non-adherence (Figure 2).

Predictors of poor adherence

On multivariable logistic regression analysis, a low educational level (OR 2.1, 95% CI 1.3-3.5, p=0.003), inadequate knowledge of HE (OR 2.8, 95% CI 1.7-4.6, p<0.001), and lactulose-related side effects (OR 1.9, 95% CI 1.2-3.1, p=0.01) were independently associated with poor medication adherence. Notably, inadequate knowledge demonstrated the strongest association. In contrast, age (per year increase; OR 1.01, 95% CI 0.99-1.02, p=0.28), male sex (OR 0.9, 95% CI 0.6-1.4, p=0.62), prior HE episode (OR 0.7, 95% CI 0.5-1.1, p=0.11), and cost concerns (OR 1.4, 95% CI 0.9-2.1, p=0.09) were not independently associated with poor adherence in the multivariable model (Table 2).

**Table 2:** Predictors of Poor Adherence to Hepatic Encephalopathy Therapy

Variable	OR (95% CI)	p-value
Low educational level	2.1 (1.3–3.5)	0.003
Inadequate knowledge	2.8 (1.7–4.6)	<0.001
Lactulose side effects	1.9 (1.2–3.1)	0.01
Age (per year increase)	1.01 (0.99–1.02)	0.28
Male sex	0.9 (0.6–1.4)	0.62
Prior HE episode	0.7 (0.5–1.1)	0.11
Cost concerns	1.4 (0.9–2.1)	0.09

## Discussion

In this study of 538 patients with cirrhosis in Bahrain, we found that knowledge of HE and adherence to therapy remain suboptimal. Despite the high prevalence of HE and its substantial clinical burden, fewer than 40% of patients demonstrated adequate understanding of the condition. These findings are consistent with prior studies reporting limited patient knowledge regarding HE pathophysiology, triggers, and management, highlighting a persistent gap in patient education across different healthcare settings [5-6,11]. Poor understanding of the disease and its treatment has been consistently associated with inadequate adherence, increased risk of HE recurrence, and higher rates of hospitalization [7,8].

Adherence to lactulose was particularly low, with only approximately half of the patients reporting good adherence. This observation aligns with real-world data demonstrating that medication utilization following HE-related hospitalization is frequently suboptimal, with a substantial proportion of patients failing to refill prescriptions within 30 days of discharge [9]. The relatively low adherence to lactulose is likely multifactorial but is predominantly driven by its unfavorable side effect profile, including diarrhea, bloating, abdominal discomfort, and the need for frequent dose titration. Studies examining barriers to lactulose adherence have consistently identified large dosing volumes, unpleasant taste, and gastrointestinal side effects as major contributors to poor compliance [4,5].

In contrast, adherence to rifaximin was notably higher, with approximately three-quarters of patients reporting good adherence. This likely reflects its favorable tolerability profile, ease of administration, and lower incidence of gastrointestinal side effects compared with lactulose. Importantly, a high proportion of patients in our cohort were prescribed rifaximin, which may reflect improved accessibility within the Bahraini healthcare system. This has important clinical implications, as combination therapy with rifaximin and lactulose has been shown to reduce HE recurrence, decrease hospitalizations, and improve survival compared with lactulose alone [2-4]. Current international guidelines recommend rifaximin as add-on therapy for secondary prophylaxis following an episode of HE [1,4], and our findings support the role of broader utilization of rifaximin, particularly in patients with poor tolerance to lactulose.

The finding that inadequate knowledge was independently associated with poor adherence (OR 2.8) underscores the critical role of patient education in the management of HE. Patients who understand the purpose of therapy, recognize early symptoms, and are aware of precipitating factors are more likely to adhere to treatment and engage in preventive behaviors [10,12]. This relationship between knowledge and adherence is well-established across chronic diseases and is particularly relevant in cirrhosis, where cognitive dysfunction may further impair self-management. Recent studies have demonstrated that structured educational interventions, including multimedia-based education and behavior change-based nursing programs, can significantly improve knowledge, medication adherence, and self-care behaviors among patients with cirrhosis [13,14]. For example, multimedia-based education has been shown to increase knowledge scores substantially within a short period, particularly in domains related to disease understanding and treatment rationale [14].

The identification of low educational level as an independent predictor of poor adherence (OR 2.1) further highlights the importance of tailoring educational strategies to patients’ health literacy. International guidelines from the American Association for the Study of Liver Diseases and the European Association for the Study of the Liver emphasize that patient and caregiver education should include clear explanations of medication purpose, expected side effects, early warning signs of HE, and appropriate actions in case of recurrence [12]. Multidisciplinary approaches involving nurses, pharmacists, and caregivers have been increasingly recognized as essential components of effective HE management and may help bridge gaps in understanding and adherence [15].

Cost was identified as a barrier to adherence in a subset of patients, although this was less prominent compared with other factors. In settings where access to medications such as rifaximin is limited, cost may represent a significant barrier to optimal therapy. However, in healthcare systems with broader medication coverage, other factors such as patient education, medication tolerability, and healthcare engagement may play a more dominant role. Importantly, the cost of pharmacological therapy should be considered in the context of the substantial economic burden associated with HE-related hospitalizations and recurrent admissions [7,8]. Interventions aimed at improving adherence, including post-discharge support, medication counseling, and telemedicine-based follow-up, have been shown to reduce readmissions and improve outcomes in patients with cirrhosis [8].

Our findings highlight several important clinical implications. First, routine assessment of patient knowledge and medication adherence should be incorporated into clinical practice for patients with cirrhosis. Second, structured educational interventions should be implemented, with particular attention to patients with low educational levels or prior non-adherence. Third, strategies to improve medication tolerability, such as optimizing lactulose dosing or early initiation of rifaximin, may enhance adherence and reduce HE recurrence. Finally, leveraging multidisciplinary care models and digital health interventions, including mobile applications and telehealth monitoring, may provide scalable solutions to improve patient engagement and long-term outcomes [10,15].

Limitations

This study has several limitations. First, the cross-sectional design precludes the ability to establish causal relationships between patient knowledge, medication adherence, and clinical outcomes. While significant associations were identified, longitudinal studies are required to determine whether improving knowledge and adherence leads to reductions in HE recurrence, hospitalizations, and mortality. Second, medication adherence was assessed using self-reported measures, which are subject to recall bias and social desirability bias. It is well established that self-reported adherence may overestimate true adherence compared with objective measures such as pharmacy refill data or medication possession ratios. Therefore, the actual rate of non-adherence in this population may be higher than reported. Third, assessment of HE knowledge was based on a structured questionnaire developed in accordance with current clinical guidelines; however, this tool has not been formally validated in external populations. Although it provides a practical and clinically relevant assessment, variability in interpretation of questions may have influenced knowledge scoring. Additionally, selection bias may be present, as patients with severe cognitive impairment were excluded, potentially underestimating true knowledge deficits. Finally, the study was conducted within Bahrain Government Hospitals, and therefore, the findings may not be fully generalizable to other healthcare systems with different patient populations, healthcare access, or medication availability. In particular, access to therapies such as rifaximin and structured follow-up may differ in other regions, potentially influencing adherence patterns.

## Conclusions

Patient knowledge and adherence to HE therapy remain suboptimal among patients with cirrhosis in Bahrain. Inadequate understanding of the disease and medication-related side effects were key barriers to adherence, with poor knowledge independently associated with non-adherence. Targeted patient education and strategies to improve treatment tolerability, including optimized use of rifaximin, are essential to improve adherence and reduce HE-related morbidity. Future studies should evaluate the impact of educational interventions on clinical outcomes such as recurrence and hospitalization.

## Appendices

Questionnaire for assessment of knowledge, adherence, and barriers in patients with cirrhosis

Section 1: Demographics

1. Age (years): ______

2. Sex: ☐ Male ☐ Female

3. Highest level of education: ☐ No formal education ☐ Primary school ☐ Secondary school ☐ University or higher

4. Are you currently working? ☐ Yes ☐ No

Section 2: Clinical Information

5. Have you been diagnosed with liver cirrhosis? ☐ Yes

6. Have you ever been diagnosed with hepatic encephalopathy (HE)? ☐ Yes ☐ No

7. If yes, how many episodes have you had? ☐ 1 ☐ 2-3 ☐ >3

Section 3: Knowledge of Hepatic Encephalopathy (Each correct answer = 1 point; total score 0-10)

8. What is hepatic encephalopathy? ☐ A brain condition caused by liver disease ☐ A heart disease ☐ A kidney problem ☐ I don’t know

9. Which of the following is a common symptom of HE? ☐ Confusion ☐ Chest pain ☐ Hair loss ☐ I don’t know

10. Which of the following can trigger HE? ☐ Constipation ☐ Infection ☐ Missing medications ☐ All of the above

11. Can HE lead to coma if untreated? ☐ Yes ☐ No ☐ I don’t know

12. What is the purpose of lactulose? ☐ Reduce toxins (ammonia) ☐ Treat blood pressure ☐ Treat diabetes ☐ I don’t know

13. What is the purpose of rifaximin? ☐ Reduce harmful gut bacteria ☐ Pain relief ☐ Treat infection in blood ☐ I don’t know

14. How often should you take HE medications? ☐ Regularly as prescribed ☐ Only when symptoms occur ☐ Only when feeling unwell ☐ I don’t know

15. What should you do if symptoms of HE worsen? ☐ Seek medical care ☐ Stop medications ☐ Ignore symptoms ☐ I don’t know

16. Can skipping medications increase risk of HE recurrence? ☐ Yes ☐ No ☐ I don’t know

17. Is HE preventable with proper treatment? ☐ Yes ☐ No ☐ I don’t know

Knowledge Score Interpretation: Adequate ≥7/10; Inadequate <7/10

Section 4: Medication Use

18. Are you currently taking lactulose? ☐ Yes ☐ No

19. Are you currently taking rifaximin? ☐ Yes ☐ No

Section 5: Medication Adherence

20. How often do you take your medications as prescribed? ☐ Always (≥80%) ☐ Sometimes (50-79%) ☐ Rarely (<50%)

21. Do you ever miss doses? ☐ Yes ☐ No

22. If yes, how often? ☐ Once per week ☐ Several times per week ☐ Frequently

Adherence Definition: Good ≥80%; Poor <80%

Section 6: Barriers to Adherence (Multiple answers allowed)

23. What are the main reasons you miss medications?

☐ Side effects (diarrhea, bloating) ☐ Do not understand purpose ☐ Cost ☐ Forgetfulness ☐ Too many medications ☐ Difficult dosing ☐ Access issues ☐ Feeling better so stopped ☐ Other: ______

Section 7: Side Effects

24. Do you experience side effects from lactulose? ☐ Yes ☐ No

25. If yes, what type? ☐ Diarrhea ☐ Bloating ☐ Abdominal pain ☐ Nausea

Section 8: Education and Counseling

26. Have you received education about HE from a doctor or nurse? ☐ Yes ☐ No

27. Would you like more information about your condition? ☐ Yes ☐ No
